# Usability of a Community-Based Dementia Resource Website: Mixed Methods Study

**DOI:** 10.2196/40762

**Published:** 2023-04-20

**Authors:** Missy Thomas, Dean Henderson, Chantal Trudel, Neil Thomas

**Affiliations:** 1 School of Industrial Design Department of Engineering and Design Carleton University Ottawa, ON Canada; 2 Bruyère Research Institute Ottawa, ON Canada; 3 The Dementia Society of Ottawa and Renfrew County Ottawa, ON Canada; 4 Department of Medicine University of Ottawa Ottawa, ON Canada

**Keywords:** dementia, caregivers, eHealth, community resources

## Abstract

**Background:**

Many individuals living with dementia want to live in their own homes for as long as possible. To do so, they frequently require assistance with activities of daily living, which is often provided by friends and relatives acting as informal care partners. In Canada, many informal care partners are currently overworked and overwhelmed. Although community-based dementia-inclusive resources are available to support them, care partners often struggle to find them. Dementia613.ca was created to make the process of finding community dementia-inclusive resources simpler and more straightforward by bringing them together in one eHealth website.

**Objective:**

The objective of our study was to determine if dementia613.ca is meeting the goal of connecting care partners and persons living with dementia to dementia-inclusive resources in their community.

**Methods:**

A review and assessment of the website was conducted using 3 evaluation methods: web analytics, questionnaires, and task analysis. Google Analytics was used to collect data related to website use over a 9-month period. Data on site content and user characteristics were collected. Furthermore, 2 web-based self-administered questionnaires were developed: one intended for care partners and persons living with dementia, and the other intended for businesses and organizations interested in serving persons living with dementia. Both gathered data on user characteristics and included standard questions used in website evaluations. Responses were collected over a 6-month period. Scenarios, tasks, and questions were developed for the moderated, remote, and task-analysis sessions. These tasks and questions determined how effectively persons living with dementia and their care partners can use dementia613.ca. Overall, 5 sessions were held with persons experiencing moderate cognitive decline and with care partners of persons living with dementia.

**Results:**

This evaluation showed that the idea behind dementia613.ca is strong and appeals to persons living with dementia, their care partners, and the businesses and organizations serving this market. Participants indicated that it is a useful community resource that meets a previously unfulfilled need in the area, and highlighted the benefits of bringing community resources together on 1 website. In our questionnaire, >60% (19/29, 66%) of people living with dementia and their care partners and 70% (7/10) of businesses and organizations agreed that the website made it easier to find relevant dementia-inclusive resources. There is room for improvement; participants indicated that the navigation and search features could be further developed.

**Conclusions:**

We believe that the dementia613.ca model could be used to inspire and guide the creation of dementia resource websites in other regions in Ontario and beyond. The framework behind it is generalizable and could be replicated to help care partners and persons living with dementia find local resources more easily.

## Introduction

### Background

In 2020, it was estimated that 597,300 Canadians were living with dementia [1]. As of 2021, there are an estimated 36,991,981 Canadians, meaning that approximately 1.6% of the population is living with dementia [1,2]. The number of Canadians with dementia will continue to rise, with 78,600 new cases being diagnosed every year in Canada [3]. By 2030, the number of people living with dementia in Canada is projected to be close to 1 million [1].

Most persons living with dementia want to live in their own homes for as long as possible, and approximately 61% of persons living with dementia in Canada are able to live at home [4,5]. Those living with dementia at home frequently require assistance with activities of daily living, which is often provided by their friends and relatives acting as informal care partners [1,6]. In Canada, informal care partners spend an average of 26 hours a week supporting their loved one with dementia [7]. Many care partners are currently either overworked or overwhelmed. The Change Foundation’s 2019 Spotlight on Caregiving Report [8] presents current data on the well-being of care partners, and many are struggling with this role. The report noted that 75% of care partners wished that there was somewhere they could go for advice. In addition, >45% of care partners of older adults with dementia exhibit symptoms of emotional distress, which is almost double the number of care partners of older adults without dementia [7].

To maintain a good quality of life, persons living with dementia must engage in physically [9] and mentally stimulating activities [9] and avoid social isolation [10]. A lack of these enriching experiences can lead to boredom, which is linked to anxiety, apathy, wandering, and agitation [11], and contributes to many of the “challenging” or “difficult” behaviors associated with dementia [12]. It can also be challenging for already overwhelmed care partners to determine the best way to provide these activities. With the many community-based dementia-inclusive resources that are available, finding the appropriate ones can be difficult or even overwhelming for care partners who are already managing multiple tasks.

Currently, there are few community-based platforms with organized and reliable information regarding dementia-inclusive resources in an easy-to-search place [13]. Web-based resource directories could provide this service and transform how persons living with dementia and care partners access information regarding community resources. Although some studies have evaluated projects that aim to connect persons with dementia and care partners with information regarding community resources [14,15], there is a lack of web-based Canadian dementia-friendly resource directories.

The website, dementia613.ca, was created to make the process of finding community dementia–inclusive resources simpler and more straightforward by bringing them together in one website. The “613” in the name of the site refers to the area code for the greater Ottawa area. Before its launch, there was no similar directory in the Ottawa and Renfrew County areas. This website was created through a partnership between The Dementia Society of Ottawa and Renfrew County (DSORC) and the Bruyère Research Institute, supported by funding from the Centre for Aging and Brain Health Innovation Spark program. There were 3 distinct phases involved in the development of dementia613.ca: phase 1, obtaining end user feedback to guide website development; phase 2, low-fidelity prototyping and participatory evaluation and design development; and phase 3, launch and evaluation. This study focuses on the third phase.

### The Creation of Dementia613.ca

Input from care partners and other stakeholder groups (eg, individuals with cognitive impairment, clinicians, and DSORC staff member) was sought during the entire design and development process. During the first phase of this project, held before the development process began, stakeholders were invited to participate in a web-based questionnaire to help inform website content and development. It was promoted to memory clinic physicians, as well as DSORC staff members, volunteers, and clients. The questionnaire included 17 questions, and responses were collected using a mixture of open-ended prompts and Likert-type scales. It gathered data on the participants’ characteristics, the types of information they wanted the website to contain, and suggestions for ease of use. Responses informed and guided website development and were referred to throughout the development process. The complete questionnaire and all the collected responses are shown in Multimedia Appendix 1. The questionnaire was available in English and all 46 respondents who started the questionnaire completed it. Most respondents identified as woman (37/46, 80%) and were aged between 45 and 64 years (25/46, 54%). Half of the respondents (23/46, 50%) identified their role as being a care partner for individual living with memory difficulties or dementia.

On the basis of the feedback gathered from these stakeholders, a website format was selected; searching on the internet was the second most common method for finding dementia-related resources. In addition, the questionnaire results indicated that the website should be mobile friendly so that it could be easily accessed on multiple types of devices. Several stakeholders indicated that they preferred to directly call the DSORC to receive information regarding resources. However, this service is only available during business hours. Stakeholder feedback provided evidence that creating a searchable web-based directory to bring existing resources together would provide a valuable service.

The second phase of this project, the development and design of the website, involved 2 rounds of participatory design testing, using 2 distinct methods. In both rounds, wireframe mock-ups of the website were created, and the participants were asked to complete simple tasks and provide feedback. Between rounds, the website design was refined, and after the second round, the final design was reached.

The first round involved unmoderated, remote tree testing. It focused on testing the overall ease of use of the website’s navigation structure and how easily the information could be found. The participants completed a series of simple tasks and questions to evaluate their ability to follow the proposed navigation logic of the website. A total of 83% (25/30) of individuals completed the study (of the participants who started the study). This round of testing was conducted for 2 weeks in May 2020. The results indicated that the overall navigation structure was efficient, and only minor adjustments were needed where participants struggled. The second round of testing involved live one-on-one task-analysis sessions. This study focused on testing the ease and efficiency of its use. During the sessions, participants were asked a series of questions and navigated the wireframe to find answers. In total, 5 individuals participated in this round of testing for over 1 week in June 2020. The results indicated that users had trouble finding and using the filter-and-sort functions of the website.

On the basis of the results of the second phase, the main features incorporated into dementia613.ca included web-based filtering tools for finding relevant resources from the database with a category search and map view, organizational listings that included specific environmental features to help users better plan a visit, information for businesses about training to become dementia-inclusive, and the ability for organizations and businesses to submit a request for their resources to be considered for inclusion on the website.

The dementia613.ca website is also fully bilingual, available in French and English, because approximately 16% of Ottawa’s population and approximately 5% of Renfrew County’s population is primarily French speaking [16].

### Goal of This Study

With the launch of demetia613.ca, this project entered its third phase, which is the focus of this study. We wanted to determine if dementia613.ca meets its goal of connecting care partners and persons living with dementia to dementia-inclusive resources in their community. The specific objective of our analysis was to evaluate feedback obtained from website users on the utility of dementia613.ca. Thus, a review and assessment of the website is required. In this paper, we present the findings from the multiple evaluation methods used during this phase, including data from web analytics, a questionnaire, and task analysis.

This study focused on evaluating website user demographics, reasons for website use, website use characteristics, and suggestions for ongoing improvement of the website.

## Methods

In phase 3 of this project, several methods were used to evaluate the launch of dementia613.ca. This included web analytics, self-administered questionnaires, and task analysis.

### Web Analytics

Google Analytics was used to collect data related to website use [17]. We collected information regarding the number of individuals who used dementia613.ca; the number of sessions (ie, the period in which a user is actively engaged with the website) that have occurred since the launch of the website; as well as demographic information regarding the visitors, geographic locations of the visitors, the frequency of the sessions, when the sessions occurred, and the top site contents viewed. Google Analytics was used to collect information from January 2021 to October 2021.

With regards to web analytics, a “session” refers to the time period users are actively engaged with the website from when they click on the link to enter the site, to when they exit the site; it includes all and any form of interaction (eg, viewing the screen or scrolling through pages on the website) per session by a single user. A page view is the number of pages viewed by a user, and the repeated views of a single page are counted.

### Self-administered Questionnaires

A total of 2 web-based, self-administered questionnaires were developed, one was intended for care partners and persons living with dementia and the other was intended for businesses and organizations interested in serving persons living with dementia. Both gathered data on users’ characteristics and included standard questions used in website evaluations. The questionnaires were reviewed and piloted internally by hospital colleagues who did not have background information on the website evaluation project.

The version for individuals collected general demographic information regarding the individual, such as gender, age, and role (role options included individual living with memory difficulties or dementia, care partner to an individual living with memory difficulties or dementia, individual who works with persons living with dementia, health care professional or other). The version for organizations collected information such as organization type and number of employees. Both versions of the questionnaire assessed satisfaction with the design, content, and navigation of the website; frequency of use; users’ perceived trust of the information; and future intentions. Responses to the questions were acquired using a Likert-type scale. In addition, there were open-ended questions for additional comments regarding the website, including reasons for use and features that users would like to see added. The complete questionnaires are shown in Multimedia Appendices 2 and 3. Both questionnaires were developed to align with others in the literature, including evaluation of eMentalHealth.ca by Jeong et al [18]. The questionnaire used in that study was based on the Commission of the European Communities’ quality criteria for health-related websites [19]. In addition, questions were added to both versions of the questionnaire to ensure that they addressed the 7 categories in the User Experience Honeycomb developed by Peter Morville [20,21], that is, useful, valuable, usable, credible, accessible, desirable, and findable. The questionnaires were customized for this study, including questions regarding the specific and unique features of the design, such as map and search functionality. Finally, the questionnaire was developed to align with previous work done in phases 1 and 2, including questions that followed up on the topics of ease of use and relevancy of information.

Questionnaire responses were collected over a 6-month period, from January 2021 to July 2021. The questionnaire was made available through a link on dementia613.ca from March 2021 to July 2021 in both French and English languages. The questionnaire was promoted on posters displayed in a Geriatric Day Hospital and Memory Clinic and in the Dementia Society Monthly Newsletter. It was also sent to the Champlain Dementia Network, a collection of organizations that support persons living with dementia, to share among their networks. To encourage participation, a draw for gift cards was done over an 8-week period.

### Task Analysis

The testing team developed a scenario, tasks, and questions for a moderated, remote, task analysis session to be held with persons experiencing moderate cognitive decline and the care partners of persons living with dementia. These tasks and questions aimed to determine how effectively persons living with dementia and their care partners could use dementia613.ca. The methods and sample sizes used to evaluate dementia613.ca aligned with the best practices in usability testing. To reach close to the user testing’s maximum benefit:cost ratio, it is standard to perform the test with 5 users per round [22-24]. This allows for multiple rounds of testing on iterations of the application, allowing designers and developers to identify and fix problems and then test the redesigned versions [25].

Tasks were created to test the 2 main features of the website (ie, web-based filtering tools and organizational listings with detailed information). The tasks centered on participants finding resources using the categories in the directory and using the map view to find resources near them. Participants who were not located in the Ottawa or Renfrew County areas were asked to complete the tasks assuming that they lived in downtown Ottawa. The tasks were based on 7 categories in User Experience Honeycomb by Morville [20,21]. To drill deeper into issues of accessibility, questions were also developed to determine whether the site content aligned with Web Content Accessibility Guidelines 2.1 [26]. Questions were designed to reach at principle 1: perceivable and principle 3: understandability [26].

The tasks and questions were reviewed and piloted internally by DSORC colleagues who did not have background information on the website evaluation project. The participants were recruited from the DSORC mailing list. The list included persons experiencing cognitive decline, current and former care partners, and DSORC dementia care coaches.

Between March 3, 2021 and March 15, 2021, five task analysis sessions, with one participant per session, were conducted. They were conducted remotely over Zoom (Zoom Video Communications, Inc) and video recorded. The participants had varying levels of experience with dementia613.ca; 3 of them had never used the site and the remaining 2 had. Sessions took between 30 and 40 minutes to complete and started with the participant accessing dementia613.ca from their own laptop or tablet and screen sharing their view with the moderator. The moderator started the task analysis by describing the scenario to the participant and then asked them to complete a series of tasks and respond to the questions. Although there were multiple ways to complete the tasks, they did have the correct end state. As such, they were marked “complete” or “incomplete” in the analysis. The time taken by each participant to complete each task was recorded. The questions were open-ended, asking about participants’ opinions and beliefs regarding the tasks and the experience of using dementia613.ca. The responses were analyzed thematically using categories from the User Experience Honeycomb of Morville [20,21].

In this scenario, participants were asked to imagine that their relatives had recently been diagnosed with dementia. The participant knew that over time, their relatives’ care needs will increase, and so the participant wanted to learn more regarding local organizations and businesses that were dementia inclusive. They planned to do this using the website dementia613.ca. For the first task, participants started on the dementia613.ca home page and were asked to find information regarding courses or programs related to the arts and crafts. The correct end-state for this task was navigating to the categories *Fitness, Exercise & Learning*. During the second task, the participants were asked to determine which listings were close to their location. To do this, they had to select *See Map View* and navigate the provided map. In the third task, the participants were asked to obtain more detailed information on an individual resource. To do this, they had to click on an individual resource, which took them to the resource’s full listing page. For the final task, participants were asked to find information regarding the organizations that run dementia613.ca. The correct end-state for this task was to find and select the *About* link. After completing the tasks, the participants were asked 4 follow-up questions to examine their overall impressions.

### Ethics Approval

The ethics application was submitted to and approved by The Bruyère Research Institute Ethics Board (# M16-19-042).

## Results

### Web Analytics

Between January and October 2021, Google Analytics reported 3924 sessions and 11,127 page views of dementia613.ca. The average length of each session was 1:40 minutes, and during that time, each user viewed an average of 2.84 pages. The list of *Resource Categories* and the French version of the site were among the top 5 viewed site features. Tables 1 and 2 and Textbox 1 summarize the web analytics collected via Google Analytics.

The vast majority of users (3143/3453, 91.02%) were new to dementia613.ca. More than two-thirds (2326/3453, 67.36%) were Canadian, and more than one-third (1212/3453, 35.1%) were located in Ottawa, Ontario. Approximately 10% (258/3453, 7.47%) were located near Ottawa, such as Gatineau, Quebec, and Ashburn, Ontario. This means that <45% (1470/3453, 42.57%) of users were in relevant locations.

Most users (62/74, 84%) accessed the website using desktop computers. In most sessions, users (2402/3924, 61.21%) reached the website directly. In other sessions, users (736/3924, 18.76%) reached the website with a referral, of which dementiahelp.ca, facebook.com, and bruyere.org were the 3 most common websites.

**Table 1:** General Google Analytics data collected from dementia613.ca from January to October 2021.

Analytic	Data collected from dementia613.ca
Timeframe	January 21, 2021 to October 28, 2021
Sessions	3924
Page views	11,127
Pages/session	2.84
Average session duration	1:51 minutes
New visitors, n (%)	3143 (91.02)
Returning visitors, n (%)	310 (9.86)

**Table 2:** Google Analytics data on device type used to access website and website traffic collected from dementia613.ca from January to October 2021.

			Values, n (%)
**Device of access to website (n=74)**			
	Desktop	62 (84)	
	Mobile	11 (15)	
	Tablet	1 (1)	
**Website traffic (n=3924)**			
	Direct	2402 (61.21)	
	Referral	736 (18.76)	
	Organic search	556 (14.17)	
	Social	228 (5.18)

**Google Analytics data on top website content viewed collected from dementia613.ca from January to October 2021.**

Top website content viewed

- /resources/
- /category/fitness-exercise-learning/
- /category/health-well–being-caregiver-services/
- /fr/acceuil/
- /category/food-beverage/
- /add-resource/
- /category/safety-monitoring/
- /category/housing-transportation/

### Self-administered Questionnaires

#### Overview

For the English and French versions of the website, 64% (39/61) of the respondents completed the questionnaire (out of the respondents who started it). Of these completed responses, 96% (28/29) of the responses were to the *Care Partner and Persons Living with Dementia Questionnaire* in English, 3% (1/29) to the *Care Partner and Persons Living With Dementia Questionnaire* in French, and 10 responses to the *Business and Organization Questionnaire* in English.

Most *Care Partner and Persons Living with Dementia* respondents (26/29, 90%) identified as woman and were aged >65 years (15/29, 52%). The majority of respondents (19/29, 66%) identified their role as being a care partner to an individual living with memory difficulties or dementia. Most *Business and Organization* respondents (6/10, 60%) worked for a health care organization.

Most *Care Partners and Persons Living With Dementia* respondents had been to dementia613.ca >5 times. The largest segment of *Business and Organization* respondents had used dementia613.ca between 2 and 4 times. Table 3 presents the results of the dementia613.ca questionnaires.

**Table 3:** Descriptive characteristics and frequency distribution for users of dementia613.ca, care partners and person living with dementia, and businesses and organizations.

Characteristics		Care partner and person living with dementia questionnaire (N=29), n (%)	Business and organization questionnaire (N-10, n (%)
**Gender**			
	Man	3 (10)	N/A^a^
	Woman	26 (90)	N/A
**Age (years)**			
	18-35	3 (10)	N/A
	36-45	3 (10)	N/A
	46-55	0 (0)	N/A
	56-64	8 (28)	N/A
	>65	15 (52)	N/A
**Role**			
	Individual living with memory difficulties or dementia	0 (0)	N/A
	Care partner to an individual with memory difficulties or dementia	19 (66)	N/A
	Individual who works with people living with dementia	5 (17)	N/A
	Health care professional	3 (10.3)	N/A
	DSORC^b^ volunteer	2 (7)	N/A
**Organization type**			
	Government	N/A	1 (10)
	Health care	N/A	6 (60)
	Other	N/A	3 (30)
**Approximate number of employees**			
	<20	N/A	4 (40)
	Between 20 and 99	N/A	3 (30)
	Between 100 and 499	N/A	1 (10)
	>500	N/A	2 (20)
**Frequency of use**			
	Once	2 (7)	1 (10)
	<5 times	8 (28)	4 (40)
	Between 5 and 9 times	10 (34)	3 (30)
	>10 times	9 (31)	2 (20)

^a^N/A: not applicable

^b^DSORC: Dementia Society of Ottawa and Renfrew County

#### Content and Ease of Use

**Figure 1:**
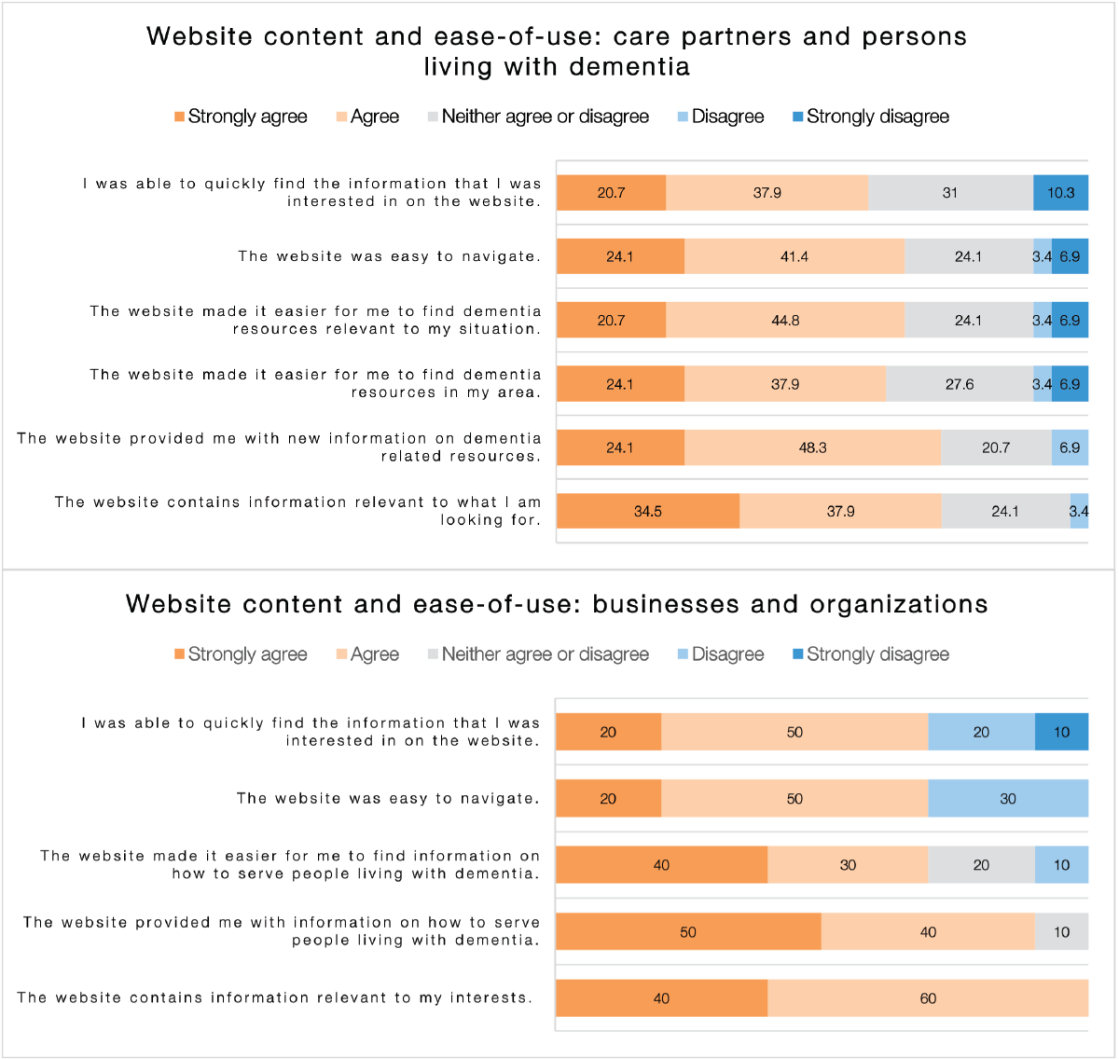
Users’ ratings of website content and ease of use of the website dementia613.ca, care partners and persons living with dementia (N=29), and businesses and organizations (N=10).

More than 70% (21/29, 72%) of the *care partners and Persons Living* respondents and 100% (10/10) of *Businesses and Organization* respondents strongly agreed or agreed that the website contained relevant information. Figure 1 presents the respondents’ ratings of the design, content, and ease of use of dementia613.ca.

#### Usefulness of Features

**Figure 2:**
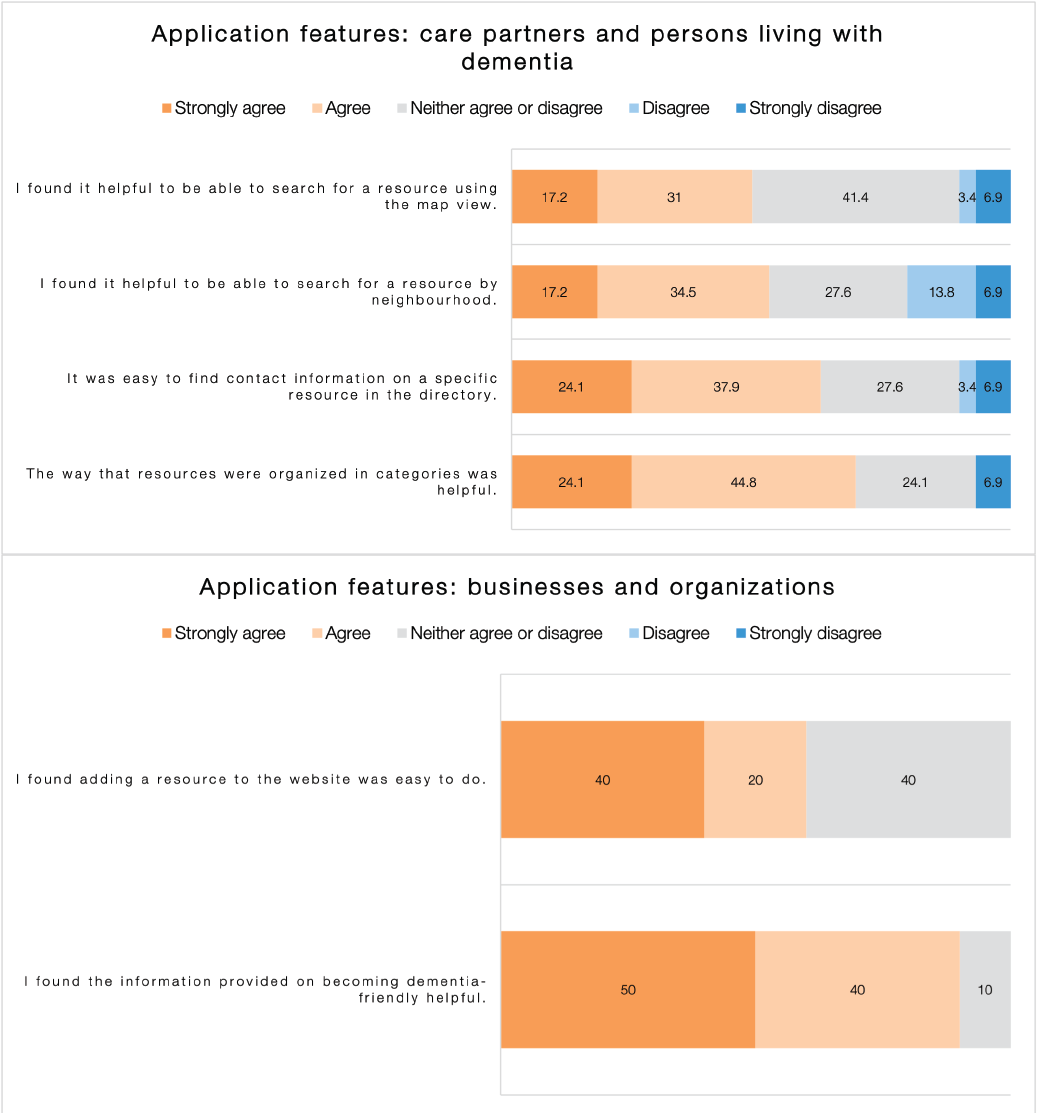
Users’ ratings of specific features of the website dementia613.ca, care partners and persons living with dementia (N=29), and businesses and organizations (N=10).

Most of the questionnaire respondents found features of dementia613.ca helpful (Figure 2). Approximately 70% (20/29, 69%) of *Care Partners and Persons Living With Dementia* respondents strongly agreed or agreed that the way the resources were organized in categories was helpful, and 90% (9/10) of *Businesses and Organizations* respondents strongly agreed or agreed that the information provided on becoming dementia friendly was helpful.

However, some features of dementia613.ca were not viewed favorably. Only 48% (14/29) of *Care Partners and Person Living with Dementia* respondents strongly agreed or agreed that it was helpful to search for resources using the map view.

#### User Satisfaction With Website

**Figure 3:**
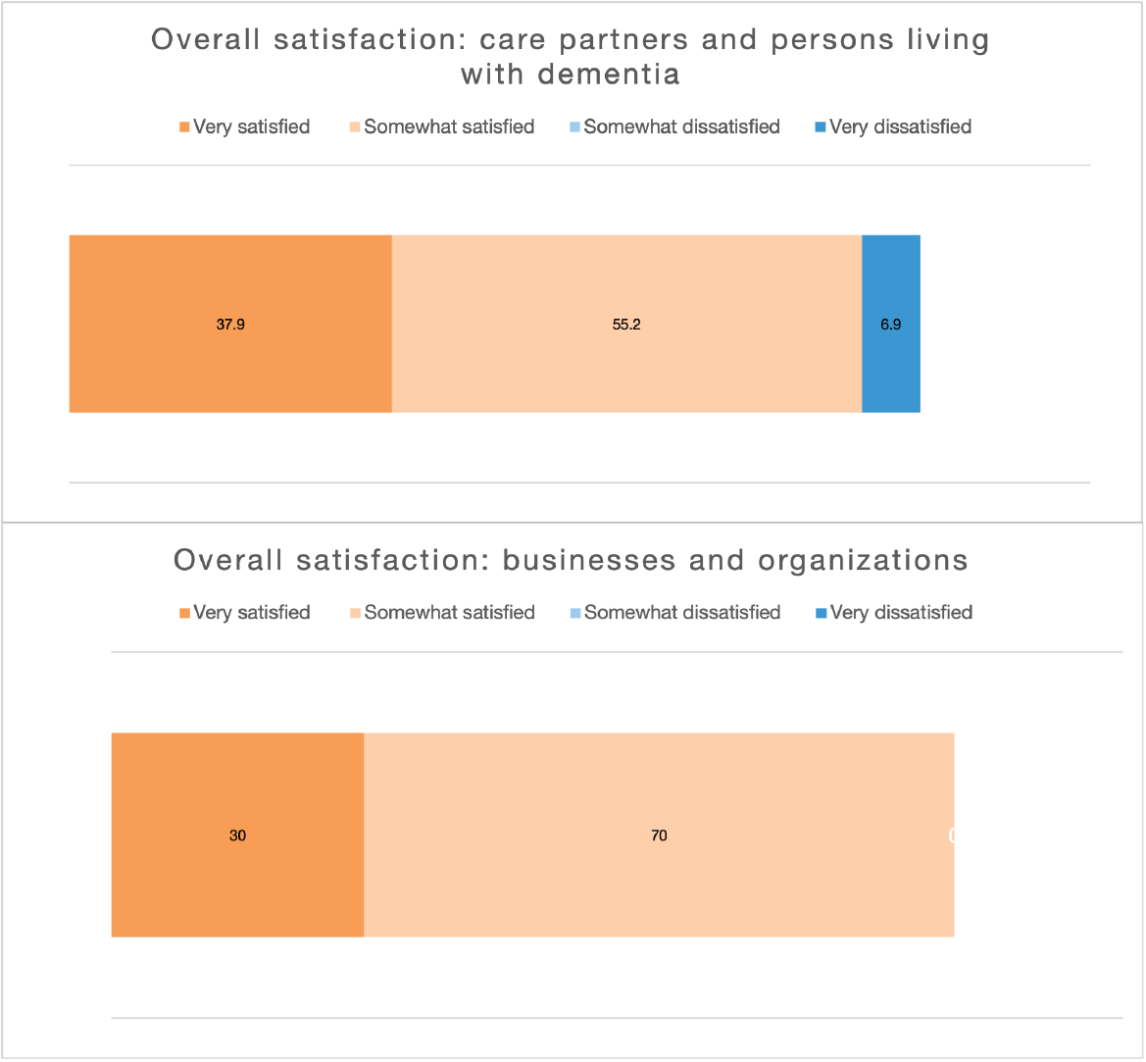
Overall satisfaction with the website dementia613.ca, care partners and person living with dementia (N=29), and businesses and organizations (N=10).

**Figure 4:**
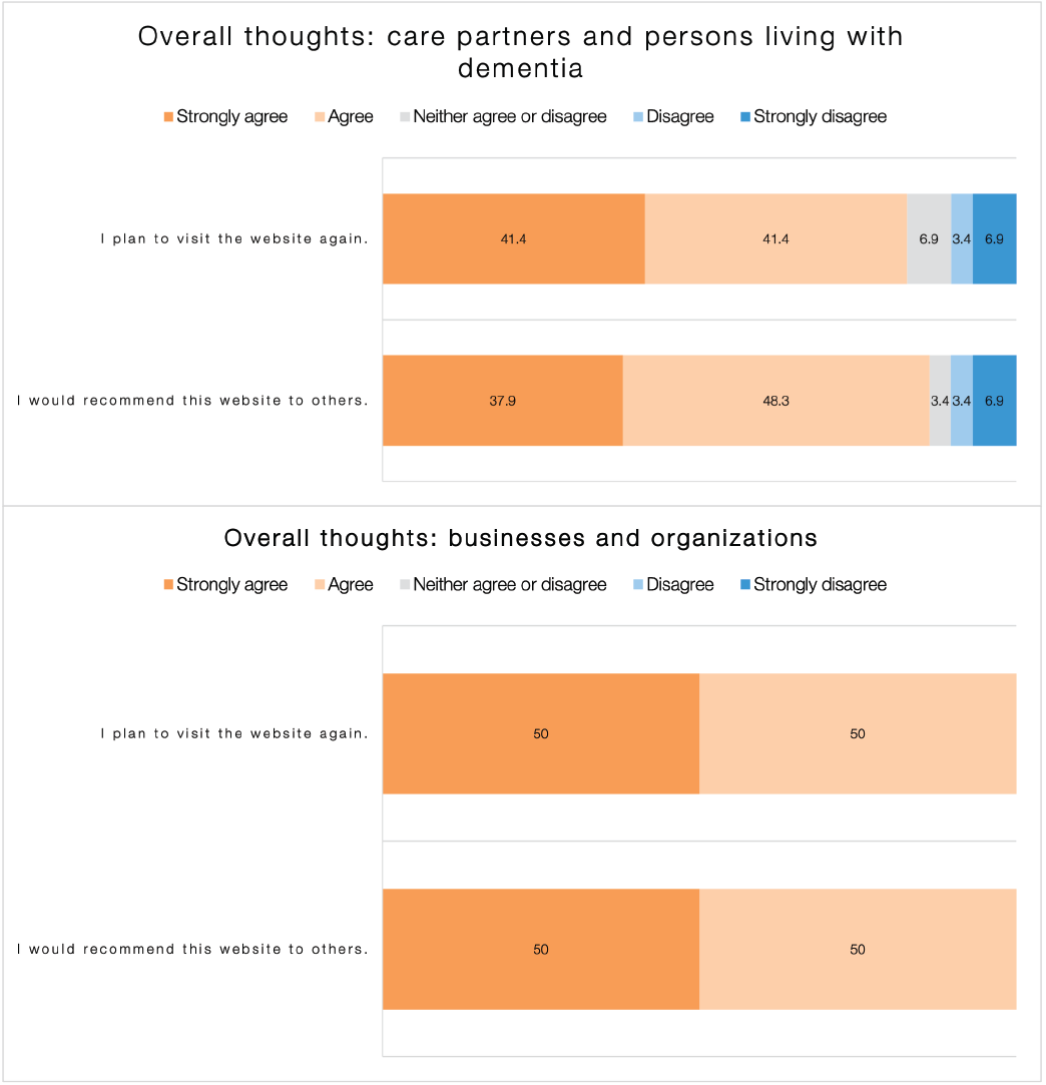
Users’ overall thoughts for the website dementia613.ca, care partners and persons living with dementia (N=29), and businesses and organizations (N=10).

As shown in Figure 3, the overall user satisfaction with dementia613.ca was very high. More than 90% (27/29, 93%) of *Care Partners and Persons Living With Dementia* respondents and 100% (10/10) of *Businesses and Organizations* respondents were very satisfied or somewhat satisfied with the website. In addition, users came away with an overall positive impression of dementia613.ca. More than 80% (24/29, 83%) of *Care Partners and Persons Living With Dementia* respondents and 100% (10/10) of *Businesses and Organizations* respondents plan to visit dementia613.ca again (Figure 4).

#### Open-ended Responses

The respondents were asked to answer specific questions at the end of the questionnaire. Descriptive coding, a type of first-cycle qualitative coding, was used to analyze the comments. In “descriptive coding,” codes are used to summarize the main topic of a passage of qualitative data [27]. This method allows responses to be grouped by theme. All the comments can be found in Multimedia Appendix 4.

Most comments were positive, stating that the website was a good resource for finding local resources, and that there was no equivalent local resource. Examples of the positive comments for each identified theme are listed in Table 4.

Negative comments were helpful because they often either critiqued specific features or offered advice on how to improve websites. Several comments noted that the lack of a search function made it challenging to find specific resources. Other comments noted that although the categories used to organize resources were helpful, the resources in each category appeared disorganized. Representative negative comments for each theme are presented in Table 5.

**Table 4:** Open-ended responses from respondents—positive and neutral comments.

Question and theme			Questionnaire type		Comment
**If you only used the website once, why did you NOT return?**					
	Newly launched resource	Business and organization Care partner and person living with dementia		“Will use more in future. Just brought to my attention.” “I did not know it existed until I saw this post.”	
**What is the reason or reason(s) you are using the website?**					
	Supporting friends and family	Care partner and person living with dementia Care partner and person living with dementia		“As a resource for education and activities for my husband.” “Looking for educational resources so I can better help my friend with dementia”	
	Supporting clients	Care partner and person living with dementia Business and organization		“To learn more about dementia friendly resources in our community that I can recommend to caregivers and people dealing with dementia.” “To send information/upcoming relevant events to members.”	
	Supporting care partners	Care partner and person living with dementia Care partner and person living with dementia		“Find resources, support groups, activities!” “Help!”	
	Supporting business development	Business and organization		“For learning purposes and to find resources on becoming more Dementia friendly.”	
	Education on dementia	Care partner and person living with dementia Care partner and person living with dementia		“Good information helps to understand illness.” “Educate myself”	
	Convince	Care partner and person living with dementia		“All available resources are together.”	
**Do you have any other thoughts you would like to share about the website?**					
	Website is helpful	Care partner and person living with dementia Care partner and person living with dementia		“This website has great potential.” “It has been very helpful for me. I have learned how to cope better. THANK YOU!”	

**Table 5:** Open-ended comments from respondents—negatives and suggestions for improvement.

Question and theme		Questionnaire type	Comment
**If you only used the website once, why did you NOT return?**			
	Hard to navigate	Care partner and person living with dementia	“No alphabetical index. No search engine. Topic categories too broad.”
	Location	Care partner and person living with dementia Care partner and person living with dementia	“Because I found it was more suited if you lived in Ottawa.” “I need a list of Memory Care facilities in [my neighborhood] but your website has none listed.”
**Are there any other features you would like us to add?**			
	Navigation features	Care partner and person living with dementia Care partner and person living with dementia	“Search engine and index.” “Search engine...and a comprehensive clickable index”
	Listing order	Care partner and person living with dementia Business and organization	“List things alphabetically in categories. It’s very scattered.” “Alphabetical order would be helpful—or maybe list the agencies that are dementia friendly first, then the others? There seems to be absolutely no logical order.”
	Categories	Care partner and person living with dementia Care partner and person living with dementia	“Make the categories less broad—under Food & Beverage, I found 211 Ontario.” “Separate out listings by region.”
	Cost information	Care partner and person living with dementia	“If there is a cost involved for example; fitness the cost should be stipulated.”
**Do you have any other thoughts you would like to share about the website?**			
	Hard to navigate	Care partner and person living with dementia Business and organization	“Search filter feature per category seems unreliable...hard to navigate to find what you’re looking for.” “The search features are not working properly.”

### Task Analysis

We conducted 5 task analysis sessions. Three participants had been care partners for a family member with dementia. Two participants had mild cognitive impairments. Furthermore, 2 of the participants were in Ottawa and the other 3 were in rural communities. Two participants self-identified as older adults (aged ≥65 years). Further details can be found in Table 6.

During the task analysis sessions, the participants were given a scenario and asked to complete 4 related tasks. The amount of time it took for users to finish the tasks was recorded, and the average successful completion time for each task is presented in Table 7. The reasons for unsuccessful task completion were as follows: in task 1 a participant did not know what category to select and gave up on the task; in task 2, a participant selected individual listings one-by-one and looked at their addresses on the full resource listing page; in task 4, a total of 2 users selected *Contact Us* instead of the correct choice *About*.

The data collected during the task analysis sessions were synthesized into takeaways based on 7 categories from User Experience Honeycomb of Morville [20,21]. Overall, most of the feedback participants provided during these sessions was positive.

Fundamentally, participants found the idea behind dementia613.ca to be good and they found the information provided to be helpful. Participants thought that the information was reliable, in large part, because of the positive reputation of the DSORC. The participants also found dementia613.ca to be accessible. The positive takeaways are listed in Table 8. However, participants also identified several areas in which dementia613.ca could be improved. The area that needs the most improvement is the overall website navigation. Users found that the organization of the website content could be improved and that specific listings were difficult to find. In addition, some users would have liked to see that business and organization listings contained more specific information. Negative and neutral takeaways are presented in Table 9.

**Table 6:** Demographic information on task analysis participants.

Code	Previously used dementia613.ca	Device	Location	Age (years)	Relation to research
P1	Yes	Desktop	Ottawa, Ontario	<65	Care partner
P2	Yes	Tablet	Renfrew, Ontario	≥65	Care partner
P3	No	Tablet	Ottawa, Ontario	≥65	Mild cognitive impairment
P4	No	Desktop	South Hampton, Ontario	<65	Mild cognitive impairment
P5	No	Desktop	South Hampton, Ontario	<65	Care partner

**Table 7:** Success rate and average completion time for tasks given to participants during task analysis sessions (n=5).

Task	Question asked	Success rate, n (%)	Average time for successful completion (min:sec)
Task 1: finding information about courses or programs related to arts and crafts	Where would you expect to find information about courses or programs related to arts and crafts?	4 (80)	1:31
Task 2: finding which listings are close to your location	How would you go about finding which listings are close to your location?	4 (80)	1:27
Task 3: finding detailed information about a resource	Once you have found a resource that interests you, how might you find out more detailed information about it?	5 (100)	0:24
Task 4: finding information about the organizations(s) that run dementia613.ca	Where would you expect to find information about the organizations(s) that run this website?	3 (60)	1:11

**Table 8:** Takeaways from task analysis session data synthesized using categories from Peter Morville’s User Experience Honeycomb—positive.

Category and key takeaways		Comments
**Useful**		
	The resources provided are relevant to user needs.	“These listings look like places I would go.”
	The full listing pages of resources provide important and relevant information.	“This page provides a good overview of information (contact, blurb, etc.).”
**Valuable**		
	Provides enough information for a user to know if a resource is relevant.	“Gives the information you need to make a decision.”
	Provides users with multiple ways to connect organizations to find more information.	“Gives enough information to know if organization is of interest and to contact them to learn more.”
**Credible**		
	Strong positive reputation of DSORC^a^.	“I assume that I can trust it because it was put there by The Dementia Society.”
	Can directly contact organizations from listings.	“I see this information as reliable. I like that there are multiple methods to contact the organization.”
**Accessibl**e		
	Content is legible.	“Content is easy to see; bold items, font size, it’s easy to read, and gives the right information.”
	Content is easy to understand.	“Wording was good, and language was understandable.”
**Desirable**		
	Concept behind dementia613.ca initiative is good.	“Overall, this could be a good resource.”
**Findable**		
	Information on resource listings is well organized.	“The listing is good and makes finding things easier.”

^a^DSORC: Dementia Society of Ottawa and Renfrew County.

**Table 9:** Takeaways from task analysis session data synthesized using categories from Peter Morville’s User Experience Honeycomb—negative or neutral.

Category	Key takeaways	Comment
Useful	The full listing pages of resources does not provide enough information.	“I would want more detailed and specific information about this resource.”
Valuable	Some resource descriptions are not specific enough.	“Some of the descriptions for the resources are not helpful. They should say exactly what the resources are.”
Usable	Overall navigation needs improvement.	“Navigation should be made easier, without so much clicking.”
Accessible	Content legibility could be improved.	“Font size needs work in places.”
Findable	Content is poorly organized.	“Content is useful, but could be organized better.”

## Discussion

### Principal Findings

Our evaluation showed that the idea behind dementia613.ca is strong and appeals to persons living with dementia and the stakeholders who serve them. Our evaluation found that although most users were new to the site, they were interested in using it again. Users saw the information on dementia613.ca as credible and felt that it provided them with relevant resources and information. Overall, the website reflects the basic ingredients needed to support a good user experience, aligning with Morville’s principles of “valuable” and “useful” [20,21].

The evaluation also identified some areas where dementia613.ca could be improved, namely navigation and search, and clarity around the website’s purpose. The needed changes will align the website with the principles of “findable” and “usable” by Morville [20,21]. We are hopeful that with these refinements, and as dementia613.ca becomes a more established and well-known resource, the site will better serve new and existing users.

### Relevance to Community

Participants shared that dementia613.ca is a useful community resource. The development process we went through to create the website involved consulting relevant members of the community (persons living with dementia, care partners, businesses, and organizations). The website was shaped by an image of their needs and desires. Our success can be seen by users citing reasons that aligned with the motivation behind its creation, such as supporting the community and providing convenient resources when asked why they were using the website. Furthermore, in task analysis sessions, several participants indicated that they saw the website as desirable, as exemplified by the comment “Overall, this could be a good resource*.*” This shows dementia613.ca’s alignment with the principle of “valuable” by Morville [20,21] by demonstrating that the website provides users with important information. Dementia613.ca is a useful resource that meets previously unmet local needs.

More specifically, dementia613.ca provided relevant resources and information to persons living with dementia and their care partners and to businesses and organizations that want to be dementia-inclusive. When asked, >60% (19/29, 66%) of persons living with dementia and their care partners and 70% (7/10) of businesses and organizations agreed that the website made it easier to find relevant dementia-inclusive resources. In open-ended responses, one participant said, “It has been very helpful for me. I have learned how to cope better. THANK YOU*!”* This shows dementia613.ca’s alignment with Morville’s principle of “useful” [20,21], demonstrating that the website serves a purpose for our stakeholders. All of this illustrates that dementia613.ca is meeting local needs by providing relevant resources and information.

Dementia613.ca is aimed at care partners, who can be of a wide variety of ages, and persons with mild cognitive impairment. It was designed to meet the needs of these users; however, we recognize that it may also be used by people outside of those demographics. To ensure that the site would be as wide as possible, we were guided by Peter Morville’s User Experience Honeycomb framework [20,21] and usability design best practices promoted by the Nielsen-Norman Group [22,23]. We also referred to Web Content Accessibility Guidelines 2.1 [26] to develop the site. Because of the principles we followed during its creation, we feel that dementia613.ca supports accessibility and the inclusion of a variety of stakeholders, recognizing that there is always room for improvement as we learn about the site in use over time.

### Areas for Improvement and Future Research

Participants provided constructive feedback that was used to produce further iterations of the design. Areas for improvement include the following:

- Refining how a website is organized as many users found it challenging to navigate. The most suggested improvement was organizing the service listings in an alphabetical order. Another suggestion is to narrow or further specify the categories provided or allow users to filter results within a category. The literature in this area aligns with these recommendations, finding that users appreciate well-designed tools to narrow search results [28]. In the next round of design and development for dementia613.ca, we will work with stakeholders to develop a more usable navigation structure. This will increase the website’s alignment with Morville’s principles of “findable” and “usable” [20,21] by improving website navigation and making it easier for users to find information they seek.
- Adding a search feature, as many users noted that the map view was not sufficient for finding specific resources. Research in this area has found that website users are goal-driven and look only for the one thing they have in mind and often rely on searching in pursuit of their goal [29]. This supports the findings of our task analysis and strengthens the case of adding a search feature. There are established best practices for developing strong on-site search engines that enable good user experience [30]. However, best practices also caution against prioritizing searches at the expense of navigation [31]. Thus, in the next round of design and development for dementia613.ca, we will work to develop both search and navigation and ensure alignment between these tools. This will further increase the website’s alignment with the principles of “findable” and “usable” [20,21].
- It is more explicit about who the website is for and what information it provides. Some users seemed unclear about the purpose of the website. In addition, a surprisingly high number of users from outside Ottawa have accessed this website. We are not sure why this occurred but it could be due to the site appearing in people’s search queries, word of mouth from people receiving the recruitment notice and passing the information onto community members outside the region, or other potential factors. This may mean that we need to clarify the purpose of the website and make it more obvious that the resources are for Ottawa and the surrounding areas.

The Google Analytics data we collected indicate that users, on average, did not spend a long time on individual pages of dementia613.ca. This could mean that individuals were looking for a specific resource or answering a specific question and were able to find the page they needed quickly. This interpretation aligns with the results from our questionnaire; approximately 60% (17/29, 59%) of the *care partners and Persons Living With Dementia* respondents strongly agreed or agreed that they were quickly able to find the information they were interested in on the website. However, short site visits could also indicate that individuals visiting the site only quickly browsed the resources before leaving, perhaps not finding useful information. Further studies are needed to understand this phenomenon. In addition, only 9% (310/3453, 8.98%) of the website visitors returned. This may be due to the timing of Google Analytics data collection, which was shortly after the launch of the website. This did not give the site much time to build a user base. Taken together, Google Analytics data brings up good points for future work to evaluate the website, which should aim to include a sample of previous website users. It should ask questions about their perceptions of the site’s utility, their primary uses for the site (using the website to find one specific resource vs using it to search for multiple different types of resources available in the area), and their reasons for returning to the site.

### Relevance to the Development of eHealth Resources

Not all web-based health resources are created equally, and the best attempts at providing useful information can fall short. To address this, there is a growing body of research on the specific information needs of internet users and their requirements for web-based health information [32]. There is also more specialized research on the barriers faced by users from marginalized populations [33,34]. A common thread among these studies is the importance of incorporating user feedback, priorities, and concerns into the design of web-based health resources. This information can be gathered by conducting evaluations with users, making it a critical step in creating useful web-based health resources.

Currently, there is a limited amount of literature evaluating public-facing health resource websites. Much of the existing research focuses on e-mental health resources [18,35-38]. These studies have found that the general public is interested in accessing quality-health resources on the web [35,39]. This aligns with our results and demonstrates the need for an increased number of reliable, public-facing health resource websites.

Other studies have found that there is a specific interest in accessing quality health resources on the web among traditionally underserved populations, including people in rural communities [40], persons with mild-intellectual disability [38,41], and adolescents and their parents [33,42]. Currently, there are scant quality web-based health resources for care partners of persons living with dementia; however, they are an overworked and overwhelmed group [7,8] that would greatly benefit from the support that this type of resource can provide. Persons living with dementia are a similarly underserved population. Dementia613.ca aims to serve both types of demographics.

Within the context of design, there is growing literature on best practices for evaluating websites in the health sector. Currently, work in this area often focuses on the websites of health institutions, such as hospitals and health departments [43-46]. This literature emphasizes the importance of working with communities and using a mixed methods approach to ensure that feedback is well-rounded and relevant [43,45]. Although dementia613.ca is a website for community resources, not for a health institution, we feel that the broader principles advanced in this body of research are aligned with these principles and approaches.

### Limitations

This study has several limitations. First, web analytics were only collected for 9 months, which is a shorter time frame than has been used in comparable research projects where the analytics have captured 12 months of data [18]. Furthermore, during the initial weeks in which web analytics were collected, final changes were made to the website design. In addition, during the data collection period, dementia613.ca was a newly launched website without an established user base. Thus, there is limited traffic to capture in the web analytics data. In future research, analytics should be conducted over a longer period. In addition, in any future evaluation, dementia613.ca will be more established, presumably with a larger flow of traffic.

The number of responses to the web-based questionnaires was small, leading to a limited sample size, especially for the *Businesses and Organizations* group. There were only 10 responses from this group, representing the health care and government sectors. This limited sample size could be viewed as homogenous. Future studies could benefit from purposive sampling to engage broader sector representation (ie, government organizations, community organizations, and private industry) to determine how well the site serves different needs and expectations among service providers.

The *Care Partners and Persons Living With Dementia* respondents skewed toward older adults and woman. Dementia613.ca was not specifically created for this demographic, suggesting that its responses may not be representative of all website users. Further research should gather feedback from a broader range of users; however, the testing done during phases 1 and 2 had responses from participants with a wide range of ages. Approximately 70% (34/46, 74%) of the responses to the phase 1 questionnaire were from individuals aged <65 years. Similarly, in phase 2, all 5 participants in the task analysis sessions were between the ages of 40 and 65 years. Thus, the site was developed using feedback from different age groups.

Furthermore, no one who self-identifies as an “individual living with memory difficulties or dementia” completed the web-based questionnaire. We promoted the study to this population through posters displayed at a Geriatric Day Hospital and Memory Clinic and in the Dementia Society Monthly Newsletter, but unfortunately did not receive any responses from this population. This limits the usability of the questionnaire results for understanding the population. However, 2 of the 5 participants in the task analysis sessions had mild cognitive impairment. Thus, the data from the task analysis sessions indicate that the initial design of dementia613.ca is appropriate for this stakeholder group. This information will be helpful to DSORC staff member when planning the future promotion of the website to both care partners and persons living with dementia.

Although Neilsen [22,23] suggested that 5 users are a reasonable number to conduct an evaluation of a website, a limited number of task analysis sessions were conducted. It has also been suggested that for qualitative studies using nonprobability sampling techniques with a homogeneous population, saturation (where potentially similar patterns emerge and no new information or insight emerges) can occur with 4-12 participants [47,48]. The small number of participants could mean that the results of the task analysis were not representative of all website users. This could be addressed in future research by reaching out to more potential participants (eg, from each stakeholder group of persons living with dementia, care partners, and businesses or organizations) and having a longer testing period. Despite this, we believe that there were enough participants to achieve saturation for the initial design of dementia613.ca.

Taken together, the amount of data collected through web analytics, questionnaires, and task analysis sessions provided enough information for an initial evaluation of the utility and ease of use of the website. The data will be used as the basis for future work aimed at improving dementia613.ca.

### Conclusions

Dementia613.ca was well received by most of the visitors. It will be a useful resource for the Ottawa area; according to the feedback we were able to collect from business owners and care partners. The participants indicated that they met a previously unfiled need in the area. Participants highlighted the utility of bringing local resources together in a web-based format in dementia613.ca. Both persons living with dementia and their care partners and businesses and organizations said that dementia613.ca provided reliable and relevant information. There is still room for improvement; participants pointed out that navigation and search features could be developed further. With further updates to the website, methods similar to those outlined in this paper will be used to obtain additional user feedback for further evaluation.

This website model can be used to inspire and guide the creation of dementia resource websites in other regions in Ontario and Canada. We believe that the framework behind dementia613.ca is generalizable and could be replicated in other regions to help care partners and persons living with dementia find local resources more easily.

## Appendix

Multimedia Appendix 1 Phase 1 survey: questions and responses.

Multimedia Appendix 2 Complete the questionnaire in English. Care partners and persons living with dementia filled out questions 1-14, and businesses and organizations filled out questions 1 and 15-26.

Multimedia Appendix 3 Complete questionnaire in French. Care partners and persons living with dementia filled out questions 1-14, and businesses and organizations filled out questions 1 and 15-26.

Multimedia Appendix 4 Questionnaire responses in English and French.

## Abbreviations

DSORC: Dementia Society of Ottawa and Renfrew County
