# Supplementary material for: Usability of a Community-Based Dementia Resource Website: Mixed Methods Study
Source: JMIR Aging. 2023 Apr 20;6:e40762. doi: 10.2196/40762 (PMC10160937; doi:10.2196/40762)

Multimedia Appendix 2. Complete the questionnaire in English. Care partners and persons living with dementia filled out questions 1-14, and businesses and organizations filled out questions 1 and 15-26.

Post-website development survey [www.dementia613.ca](http://www.dementia613.ca/) Dementia Friendly Website Feedback Survey

**This is a study about helping caregivers and people living with dementia ﬁnd dementia friendly resource programs in their area. Lots of programs are available, but you may not be aware of their availability and accessibility. As part of the study we are trying to understand the steps that caregivers and people living with dementia take in trying to ﬁnd and access dementia friendly resources.**

**Please take 5-10 minutes to complete the survey and provide us with your feedback on our website of Dementia friendly resources in your area.**

**No identifying information will be collected. The survey is anonymous and your participation is voluntary.**

**This survey is administered by SurveyMonkey. The SurveyMonkey server is housed in the United-States. If you agree to participant please proceed with answering the following questions.**

**Thank you for your time and support!**

- 1. How would you describe yourself?

I am an individual looking for dementia related resources (e.g., I am a care partner to an individual living with memory diﬀiculties or dementia, an individual living with memory diﬀiculties or dementia, an individual who works with people living with dementia, or a health care professional).

I am part of an organization interested in serving people living with dementia through my business or organization.

Post-website development survey [www.dementia613.ca](http://www.dementia613.ca/)

- 2. What is your role?

Care partner to an individual living with memory diﬀiculties or dementia Individual living with memory diﬀiculties or dementia

Health Care Professional (Please specify below) Individual who works with people living with dementia Other (Please specify below)

Please specify here

* 3. What is your age category?

18-35

36-45

46-55

56-64

65+

- 4. What is your gender?

Female Male

Prefer not to answer Other (please specify)

- 5. How often did you use the website to look for Dementia friendly resources?

Once

Less than 5 times Between 5-9 times More than 10 times

6. If you used the website once, why did you NOT return?


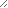


- 7. Please rate the following statements about website content and ease-of-use.

Strongly agree Agree

Neither agree or

disagree Disagree

Strongly disagree

The website contains information relevant to what I am looking for.

The website provided me with new information on dementia related resources.

The website made it easier for me to ﬁnd dementia resources in my area.

The website made it easier for me to ﬁnd dementia resources relevant to my situation.

The website was easy to navigate.

I was able to quickly ﬁnd the information that I was interested in on the website.

- 8. Please rate the following statements about website application features.

Strongly agree Agree

Neither agree or

disagree Disagree

Strongly disagree

The way that resources were organized in categories was helpful.

It was easy to ﬁnd contact information on a speciﬁc resource in the directory.

I found it helpful to be able to search for a resource by neighbourhood.

I found it helpful to be able to search for a resource using the map view.

* 9. Overall, how satisﬁed are you with the website?

Very satisﬁed Satisﬁed Dissatisﬁed

Very dissatisﬁed

- 10. Please rate the following statements about your overall thoughts

Strongly agree Agree

Neither agree or

disagree Disagree

Strongly disagree

I would recommend this website to others.

I plan to visit the website again.

1. What is/are the reason(s) you are using the website?


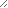


1. Can you think of any websites that are similar to this one?


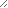


1. Are there any other features you would like us to add?


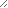


1. Do you have any other thoughts you would like to share about the website?


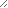


Post-website development survey [www.dementia613.ca](http://www.dementia613.ca/)

* 15. How would you describe your organization?

Cultural (Museum, Historical site, etc.) Community (Library, School, etc.) Religious

Government Charity Healthcare Retail Restaurant

Other (please specify)

- 16. Approximately how many employees do you have?

Less than 20 employees Between 20 and 99 employees

Between 100-499 employees 500 or more employees

- 17. How often did you use the website?

Once

Less than 5 times Between 5-9 times More than 10 times

18. If you used the website once, why did you NOT return?


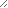


- 19. Please rate the following statements about website content and ease-of-use.

Strongly agree Agree

Neither agree or

disagree Disagree

Strongly disagree

The website contains information relevant to my interests.

The website provided me with information on how to serve people living with dementia

The website made it easier for me to ﬁnd information on how to serve people living with dementia.

The website was easy to navigate.

I was able to quickly ﬁnd the information that I was interested in on the website.

- 20. Please rate the following statements about website application features.

Strongly agree Agree

Neither agree or

disagree Disagree

Strongly disagree

I found the information provided on becoming dementia-friendly helpful.

I found adding a resource to the website was easy to do.

* 21. Overall, how satisﬁed are you with the website?

Very satisﬁed Satisﬁed Dissatisﬁed

Very dissatisﬁed

- 22. Please rate the following statements about your overall thoughts

Strongly agree Agree

Neither agree or

disagree Disagree

Strongly disagree

I would recommend this website to others.

I plan to visit the website again.

1. What is/are the reason(s) you are using the website?


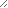


1. Can you think of any websites that are similar to this one?


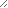


1. Are there any other features you would like us to add?


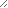


1. Do you have any other thoughts you would like to share about the website?


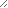

Supplement: Multimedia Appendix 2 [file aging_v6i1e40762_app2.docx]
