# Supplementary material for: Usability of a Community-Based Dementia Resource Website: Mixed Methods Study
Source: JMIR Aging. 2023 Apr 20;6:e40762. doi: 10.2196/40762 (PMC10160937; doi:10.2196/40762)
Supplement: Multimedia Appendix 3 [file aging_v6i1e40762_app3.docx]

Multimedia Appendix 3. Complete questionnaire in French. Care partners and persons living with dementia filled out questions 1-14, and businesses and organizations filled out questions 1 and 15-26.

Sondage suivant le lancement du site internet [www.dementia613.ca](http://www.dementia613.ca/) Questionnaire sur l'application des ressources sur la démence

# Cette étude vise à aider les personnes atteintes de démence et leurs aidants à trouver des programmes adaptés dans leur région. De nombreux programmes sont disponibles, mais vous ne connaissez peut-être pas leur disponibilité et leur accessibilité. Dans le cadre de ce sondage, nous essayons de comprendre les étapes eﬀectuées par les personnes atteintes de démence et leurs aidant pour trouver et accéder à des ressources adaptées à la démence.

**Veuillez s’il vous plait prendre 10 à 15 minutes pour répondre au questionnaire et nous faire part de vos commentaires sur notre application mobile de ressources adaptées à la démence dans votre région.**

**Aucune information d'identiﬁcation ne sera collectée. Le questionnaire est anonyme et votre participation est volontaire.**

**Ce questionnaire est administrée par SurveyMonkey. Le serveur SurveyMonkey est hébergé aux États-Unis. Si vous acceptez de participer, veuillez s’il vous répondre aux questions suivantes.**

**Merci pour le temps de répondre à ce questionnaire et pour votre soutien!**

## * 1. Comment vous décririez-vous?

Je suis une personne à la recherche de ressources liées à la démence (p. ex., je suis le/la partenaire de soins d'une personne vivant avec des problèmes de mémoire ou ayant une démence, une personne vivant avec

des problèmes de mémoire ou une démence, une personne qui travaille avec des personnes atteintes de démence, ou un professionnel de la santé).

Je fais partie d'une organisation intéressée à servir les personnes atteintes de démence par le biais de mon entreprise ou de mon organisation.

Sondage suivant le lancement du site internet [www.dementia613.ca](http://www.dementia613.ca/)

## * 2. Quel est votre rôle?

Partenaire de soins d'une personne vivant avec des troubles de la mémoire ou une démence Personne vivant avec des troubles de la mémoire ou une démence

Professionnel de la santé (Veuillez s’il vous plaît préciser) Personne qui travaille avec des personnes atteintes de démence Autre (Veuillez s’il vous plaît préciser)

Veuillez s’il vous plaît préciser

## * 3. Quel est votre groupe d’âge ?

18-35

36-45

46-55

56-64

65+

## * 4. Quel est votre genre ?

Masculin Féminin

Préfère ne pas répondre

Autre (Veuillez s’il vous plaît préciser )

## * 5. À quelle fréquence avez-vous utilisé le site internet pour rechercher des ressources adaptées à la démence?

Une fois

Moins de 5 fois Entre 5 et 9 fois Plus de 10 fois

## 6. Si vous avez utilisé le site internet qu'une seule fois, pourquoi n'êtes-vous pas revenu?


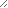


* 7. Veuillez s’il vous plaît indiquer si vous êtes en accord ou désaccord avec les énoncés suivants.

Tout à fait

d'accord D'accord

Ni d'accord ni en

désaccord Pas d'accord

Pas du tout d'accord

Le site internet contient des informations pertinentes à ce que je recherche.

Le site internet m'a fourni de nouvelles informations sur les ressources liées à la démence.

Le site internet m'a permis de trouver plus facilement des ressources sur la démence dans ma région.

Le site internet m'a permis de trouver plus facilement des ressources sur la démence pertinentes à ma situation

Le site internet était facile à naviguer

J'ai pu trouver rapidement les informations qui m'intéressaient sur le site.

## 8. Fonctionnalités de l'application

Tout à fait

d'accord D'accord

Ni d'accord ni

en désaccord Pas d'accord

Pas du tout d'accord

La façon dont les ressources étaient organisées en catégories a été utile.

Il était facile de trouver les coordonnées d'une ressource spéciﬁque dans l'annuaire.

J'ai trouvé utile de pouvoir rechercher une ressource par quartier.

J'ai trouvé utile de pouvoir rechercher une ressource en utilisant la carte.

## * 9. Dans l'ensemble, dans quelle mesure êtes-vous satisfait du site internet ?

Très satisfait Plutôt satisfait Plutôt insatisfait Très insatisfait

## 10. Veuillez noter les aﬀirmations suivantes concernant vos pensées générales

Tout à fait

d'accord D'accord

Ni d'accord ni en

désaccord Pas d'accord

Pas du tout d'accord

Je recommanderais ce site internet à d'autres

Je prévois de visiter à nouveau le site interne

## Pour quelle (s) raison (s) utilisez-vous le site internet ?


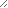


1. Connaissez-vous des sites internet similaires à celui-ci?


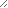


1. Y a-t-il d'autres fonctionnalités que vous aimeriez que nous ajoutions?


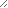


1. Avez-vous d'autres idées que vous aimeriez partager au sujet de ce site ?


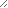


Sondage suivant le lancement du site internet [www.dementia613.ca](http://www.dementia613.ca/)

* 15. Comment décririez-vous votre organisation ?

Centrée sur la culture (musée, site historique, etc.) Communautaire (bibliothèque, école, etc.) Religieuse

Gouvernementale Caritative

Dans le domaine de la santé

Dans le domaine de la vente au détail Dans le domaine de la restauration Autre (veuillez s’il vous plaît préciser)

## 16. Combien d'employés comptez-vous environ?

Moins de 20 employés Entre 20 et 99 employés

Entre 100 et 499 employés 500 employés ou plus

## 17. À quelle fréquence avez-vous utilisé le site internet ?

Une fois

Moins de 5 fois Entre 5 et 9 fois Plus de 10 fois

## 18. Si vous avez utilisé le site internet qu'une seule fois, pourquoi n'êtes-vous pas revenu?


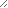


- 19. Veuillez s’il vous plaît indiquer si vous êtes en accord ou désaccord avec les énoncés suivants.

Tout à fait

d'accord D'accord

Ni d'accord ni en

désaccord Pas d'accord

Pas du tout d'accord

Le site contient des informations relatives à mes intérêts.

Le site m'a fourni des informations sur la façon de servir les personnes atteintes de démence

Le site m'a permis de trouver plus facilement des informations sur la façon de servir les personnes atteintes de démence.

Le site était facile à naviguer.

J'ai pu trouver rapidement les informations qui m'intéressaient sur le site.

## 20. Veuillez s’il vous plaît indiquer si vous êtes en accord ou désaccord avec les énoncés suivants.

Tout à fait

d'accord D'accord

Ni d'accord ni en

désaccord Pas d'accord

Pas du tout d'accord

J'ai trouvé utile les informations fournies sur la façon de mieux accueillir les personnes atteintes de démence.

J'ai trouvé qu’il était facile d'ajouter des ressource au site.

## * 21. Dans l'ensemble, dans quelle mesure êtes-vous satisfait du site internet ?

Très satisfait Plutôt satisfait Plutôt insatisfait Très insatisfait

## 22. Veuillez s’il vous plaît indiquer si vous êtes en accord ou désaccord avec les énoncés suivants.

Tout à fait

d'accord D'accord

Ni d'accord ni en

désaccord Pas d'accord

Pas du tout d'accord

Je recommanderais ce site internet à d'autres

Je prévois de visiter à nouveau le site internet

## Pour quelle (s) raison (s) utilisez-vous le site internet ?


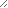


1. Connaissez-vous des sites internet similaires à celui-ci?


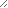


1. Y a-t-il d'autres fonctionnalités que vous aimeriez que nous ajoutions?


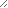


1. Avez-vous d'autres idées que vous aimeriez partager au sujet de ce site?


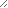


Merci pour vos commentaires et pour avoir répondu à notre questionnaire!
